# Impact of Depressive Disorder on Periodontal Status: A Comparative Study

**DOI:** 10.3390/dj13090429

**Published:** 2025-09-16

**Authors:** Bogdan-Constantin Vasiliu, Maria Alexandra Mârțu, Alexandra Cornelia Oanță, Irina Șufaru, Liliana Păsărin, Alexandru Ionuț Luchian, Sorina Mihaela Solomon

**Affiliations:** Department of Periodontology, Faculty of Dental Medicine, “Grigore T. Popa” University of Medicine and Pharmacy, 700115 Iasi, Romania; bogdan.vasiliu@umfiasi.ro (B.-C.V.); alexandra-cornelia.c.oanta@umfiasi.ro (A.C.O.); ursarescu.irina@umfiasi.ro (I.Ș.); liliana.pasarin@umfiasi.ro (L.P.); ionut.luchian@umfiasi.ro (A.I.L.); sorina.solomon@umfiasi.ro (S.M.S.)

**Keywords:** periodontitis, full-mouth disinfection, depression, selective serotonin reuptake inhibitors, probing depth, clinical attachment level, plaque index, bleeding on probing

## Abstract

**Background/Objectives**. Periodontitis is a chronic inflammatory disease influenced by systemic and psychological factors, including depression. Selective serotonin reuptake inhibitors (SSRIs), widely used to treat depression, may also affect periodontal healing. This study aimed to evaluate the clinical efficacy of full-mouth disinfection (FMD) in patients with periodontitis, with or without comorbid depression and SSRI therapy. **Methods**. Eighty participants were enrolled and divided into two groups: periodontitis only (n = 40) and periodontitis with depression (n = 40), the latter subgrouped by SSRI usage. Clinical parameters, including probing depth (PD), clinical attachment level (CAL), bleeding on probing (BOP), and plaque index (PI), were assessed at baseline and 12 weeks after FMD. **Results**. Following FMD, significant improvements were observed in PD, PI, and BOP across all groups (*p* < 0.001). In the non-depressed group, mean PD decreased from 4.26 ± 0.97 mm to 2.76 ± 0.56 mm (*p* < 0.001) and PI from 3.85 ± 0.70 to 1.05 ± 0.99. Patients with depression had higher initial PD (4.98 ± 1.05 mm) but still showed improvement to 3.08 ± 0.69 mm (*p* < 0.001). CAL improved significantly only in non-depressed individuals (*p* = 0.008), while no statistically significant CAL changes were observed in depressed patients (*p* > 0.05). SSRI therapy did not significantly influence treatment outcomes (*p* > 0.05). **Conclusions**. FMD is clinically effective in reducing periodontal inflammation in patients with or without depression. However, improvements in CAL were more pronounced in non-depressed individuals, suggesting that depression may partially attenuate periodontal healing.

## 1. Introduction

Periodontal disease represents a persistent inflammatory disorder primarily driven by a dysbiotic shift in the oral microbiome. Clinically, it manifests through symptoms such as gingival inflammation, bleeding upon probing, progressive loss of periodontal attachment, and increased tooth mobility. The course of the disease is largely influenced by the dynamic interplay between the host’s immune system and microbial factors, where an imbalance (whether due to microbial overgrowth or impaired immune regulation) can accelerate the destruction of the tissue [1]. Pathogenic bacterial species associated with periodontitis stimulate a cytokine-driven inflammatory response. This process compromises the integrity of the gingival soft tissues and promotes alveolar bone resorption by enhancing osteoclast activity [2].

Risk factors for periodontal disease include direct contributors such as smoking, systemic illnesses, poor oral hygiene, and nutritional status. Indirect factors include age, genetic predisposition, iatrogenic influences, and psychological stress [3,4]. Accurate diagnosis requires a thorough periodontal evaluation, focusing on site-specific clinical parameters such as probing depth (PD), clinical attachment level (CAL), bleeding on probing (BOP), and plaque index (PI) [5].

Each case of periodontitis is shaped by a patient’s unique oral microbial ecology and individualized risk profile, leading to significant variability in disease expression. Without timely treatment, the disease may progress, leading to the gradual breakdown of periodontal structures and possible tooth loss. Nonsurgical therapeutic approaches remain the primary modality for restoring host–microbial balance, with scaling and root planing (SRP) regarded as the standard of care [6,7]. However, the success of such interventions may be undermined by systemic illnesses, tobacco consumption, or mental health conditions, such as depression, which have been shown to negatively impact treatment outcomes [8].

To enhance outcomes, alternative techniques such as quadrant-based debridement (Q-SRP), full-mouth scaling (FMS), and full-mouth disinfection (FMD) have been developed. These aim to reduce recolonization by simultaneously treating all intraoral sites [9]. Adjunctive methods, including ultrasonic debridement, glycine powder air-polishing (FMDAP), and laser or photodynamic therapy, have further optimized these protocols [10,11].

The increasing prevalence of depression, marked by anhedonia, hopelessness, and reduced engagement, has drawn attention to its potential influence on periodontal health [12]. Behavioral factors such as poor hygiene or smoking, comorbidities such as diabetes and chronic stress have been linked to greater periodontal severity [13,14]. Neuroendocrine–immune mechanisms likely mediate this association, with elevated inflammatory responses observed under psychological distress [15,16].

Selective serotonin reuptake inhibitors (SSRIs) widely used as first-line pharmacological agents in the treatment of depression, function by enhancing serotonergic activity in the central nervous system. Despite their therapeutic benefits, SSRIs have been associated with several adverse effects, including xerostomia and altered immune function. They may also cause disturbances in bone homeostasis, such as suppressed osteogenesis and increased bone resorption [17,18]. These pharmacological effects have raised concerns regarding their potential influence on periodontal health and treatment outcomes.

The study aimed to assess the clinical efficacy of full-mouth disinfection (FMD) procedure in patients with periodontitis, with or without comorbid depressive disorder and SSRI treatment, by evaluating changes in PD, BOP, CAL, and PI. The null hypothesis stated that FMD would not produce significant improvements in these clinical parameters, regardless of the presence of depressive symptoms or SSRI medication.

## 2. Materials and Methods

### 2.1. Study Design and Population

A prospective observational study with intervention (FMD therapy) was conducted to evaluate periodontal parameters in patients with and without depressive disorders. The sample size was calculated using G*Power software version 3.1 (Heinrich Heine University, Düsseldorf, Germany), based on an effect size of 0.462, α = 0.05, and a power of 0.8, resulting in a minimum of 80 participants. Data collection was performed between 15 February 2024 and 15 May 2024. The study was conducted in accordance with the Declaration of Helsinki, and approved by the Ethics Committee of Grigore T. Popa University of Medicine and Pharmacy (protocol code 400/15 February 2024).

The study population included 80 participants, divided equally into two groups:

Group P (n = 40): patients with clinically diagnosed periodontitis, presence of Ramfjord teeth, and adequate cooperation.

Group D (n = 40): patients diagnosed with both periodontitis and depressive disorder (with or without antidepressant therapy), also presenting Ramfjord teeth and cooperative behavior.

Each group was subdivided into initial evaluation and reevaluation subgroups, as detailed in Table 1.

Additionally, comparative analyses were conducted between gender-based subgroups, male (m) vs. female (f), within each experimental condition (e.g., Pi, Pr, Di, Dr) to evaluate potential gender-specific differences in clinical outcomes. In group P the distribution of the sexes was 18 males and 22 females, while in group D, there were 19 males and 21 females.

Moreover, group D was further divided into two subgroups (consisting of subjects receiving SSRI medication (DM) and subjects not receiving SSRI medication (D0)). Both subgroups were initially assessed (DiM and Di0, respectively) and reassessed at the end of FMD therapy (DrM and Dr0, respectively). Among the 40 patients with depression, 24 were undergoing treatment with selective serotonin reuptake inhibitors (SSRIs), while 16 were untreated.

**Figure 1:**
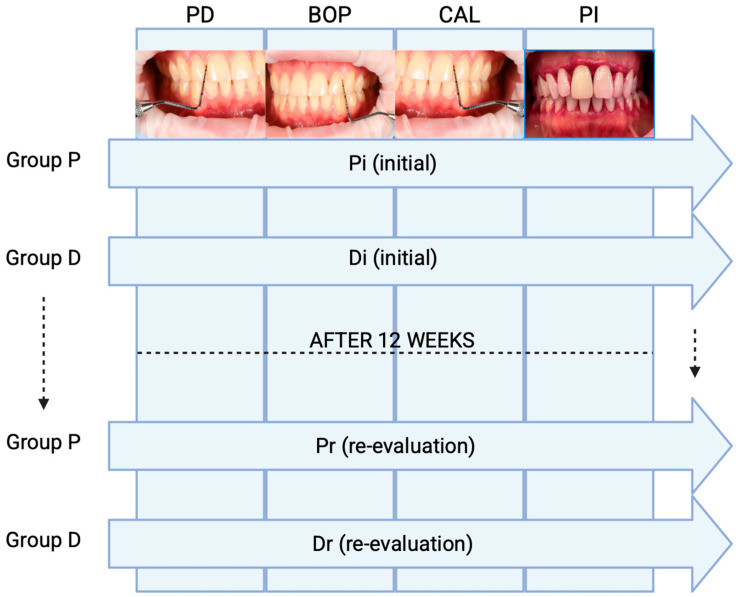
The study design. Abbreviations: Pi = periodontitis initial; Pr = periodontitis reevaluation; Di = depression initial; Dr = depression reevaluation; PD = Probing Depth; CAL- Clinical Attachment Loss; PI- Plaque Index; BOP- Bleeding on Probing.

The study design is presented in Figure 1.

### 2.2. Clinical Assessment Protocol

All participants underwent a baseline clinical examination, including periodontal diagnosis by visual inspection and palpation.

Periodontal parameters assessed were

- Probing Depth (PD)
- Clinical Attachment Loss (CAL)
- Modified Quigley Hein Plaque Index (PI)
- Bleeding on Probing (BOP)

PD and CAL were measured at six sites per Ramfjord tooth (1.6, 2.1, 2.4, 3.6, 4.1, 4.4), using the cementoenamel junction and the gingival margin as reference points. CAL was calculated as the sum of pocket depth and gingival recession.

The plaque index (QH), modified by Turesky [19], was recorded after plaque disclosure. Buccal and oral surfaces of Ramfjord teeth were scored from 0 (no plaque) to 5 (plaque covering more than two-thirds of the crown). The final score was calculated as the mean of all surfaces evaluated.

BOP was measured by probing four sites per Ramfjord tooth. The index was expressed as the percentage of bleeding surfaces out of a total of 24.

Prior to data collection, two examiners were calibrated using 10 volunteer patients not included in the study. Measurement reliability was assessed using the Intraclass Correlation Coefficient (ICC), and both examiners achieved an ICC ≥ 0.85, indicating excellent agreement. Patients were not blinded to treatment allocation due to the nature of the intervention.

### 2.3. Therapeutic Intervention

All participants received full-mouth disinfection (FMD) within 24 h, following a standardized protocol (Table 2):

Initial Phase:

– Clinical evaluation (PD, CAL, BOP)
– Collection of saliva and crevicular fluid
– Oral hygiene instruction and motivation
– Supragingival mechanical debridement

FMD Protocol (repeated per quadrant):

– Supraperiosteal local anesthesia
– Subgingival instrumentation using ultrasonic tips (P3) and Gracey Micro Mini Five curettes
– Antiseptic rinsing with 0.2% chlorhexidine and 3% hydrogen peroxide

Adjunctive Therapy:

– 0.2% chlorhexidine rinses, 2–3 times daily for 14 days
– Where indicated, systemic antibiotics: Augmentin 500 mg/8 h and Metronidazole 250 mg/8 h

Reevaluation was performed at 12 weeks post-treatment using identical clinical methods.

### 2.4. Statistical Analysis

The statistical analysis was conducted using IBM SPSS Statistics 29.0.0 software.

Normality of distribution was assessed using Shapiro–Wilk tests while Levene’s test was used to evaluate the homogeneity of variances. Group comparisons were performed using: Parametric tests: One-way ANOVA with Bonferroni post hoc and non-parametric test Kruskal–Wallis. A significance level of *p* < 0.05 was used throughout the analyses.

## 3. Results

### 3.1. Probing Depth (PD)

**Figure 2:**
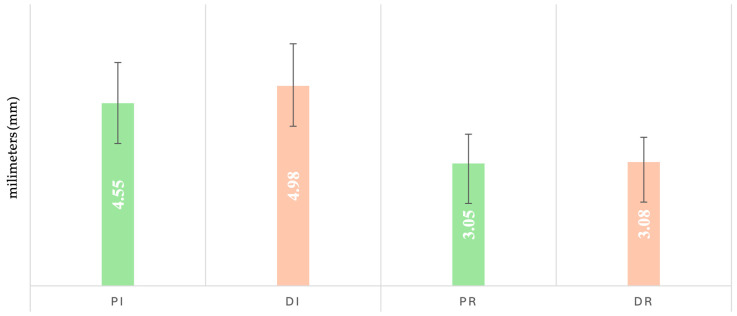
Changes in probing depth (PD) at baseline and 12 weeks after full-mouth disinfection.

At baseline (initial), the highest PD mean was observed in the depression group (Di: 4.98 ± 1.05), which also maintained the highest value at reevaluation (3.08 ± 0.69) (Figure 2). Normality tests indicated non-Gaussian distributions in Pi and Di subgroups. Levene’s test confirmed variance heterogeneity (*p* < 0.001), prompting the use of the Kruskal–Wallis test. Statistically significant differences were found between Di vs. Dr, Di vs. Pi, and Pr vs. Pi (*p* < 0.001; Bonferroni-adjusted).

Gender-based analysis showed higher baseline PD in Di M (5.2 ± 1.2) compared to Di F (4.7 ± 0.8). Significant differences were identified between Di (F) vs. Dr (F); Di (F) vs. Pi (F); Di (M) vs. Dr (M); Di (M) vs. Pi (M); Pi (F) vs. Pr (F); and Pi (M) vs. Pi (F) (*p* < 0.01).

Regarding the use of specific medication, the patients without SSRI medication showed higher PD values both initially (5.1 ± 1.2) and at reevaluation (3.1 ± 0.5). Variance analysis required nonparametric comparison, with significant differences between Di0 vs. Dr0 and DiM vs. DrM (*p* = 0.000).

### 3.2. Clinical Attachment Loss (CAL)

**Figure 3:**
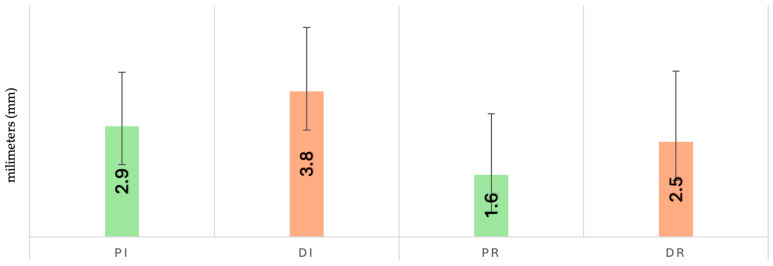
Changes in clinical attachment level (CAL) at baseline and 12 weeks after full-mouth disinfection.

The Di group recorded the highest initial CAL values (3.8 ± 1.7), while Dr had the highest at reevaluation (2.5 ± 1.8) (Figure 3). Shapiro–Wilk testing indicated non-normal distribution in Pi and Pr, and Dr. Levene’s test confirmed variance homogeneity (*p* > 0.05), allowing parametric testing.

One-way ANOVA revealed significant differences (F = 6.042; *p* < 0.001), and Bonferroni post hoc testing showed significance only between Pi and Pr (*p* = 0.008). No statistically significant differences were observed between sex-based subgroups (*p* = 0.059).

In the depression group, the highest initial CAL value was recorded in the SSRI-treated subgroup (3.9 ± 1.9), while the highest reevaluation value was noted in the untreated subgroup (2.6 ± 1.7). No statistically significant differences were found (ANOVA, *p* = 0.112).

### 3.3. Bleeding on Probing (BOP)

**Figure 4:**
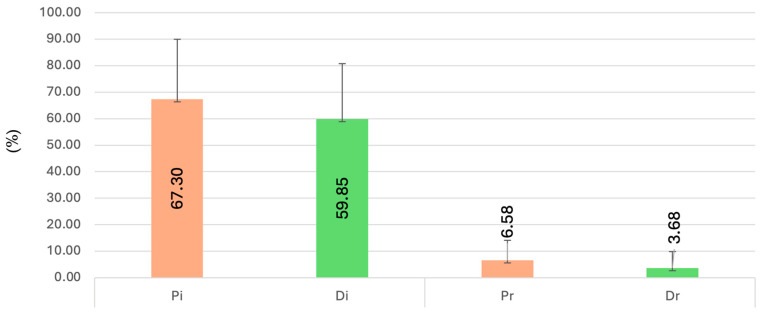
Changes in bleeding on probing (BOP) at baseline and 12 weeks after full-mouth disinfection.

The Pi subgroup showed the highest initial BOP (67.30 ± 22.66), while Pr had the highest value at reevaluation (6.58 ± 7.58) (Figure 4).

Due to heterogeneity of variances (*p* < 0.001), Kruskal–Wallis testing was applied. Statistically significant differences were found between Dr vs. Pr and Dr vs. Di (*p* < 0.001). In the sex-based subgroup analysis, significant differences were found between Dr (F) vs. Pr (F), Dr (F) vs. Di (F), Dr (M) vs. Pr (M), and Dr (M) vs. Di (M) (*p* < 0.05).

Within the depression group, untreated patients showed the highest baseline BOP (68.0 ± 21.1), while the SSRI-treated subgroup had the highest value at reevaluation (4.5 ± 6.9). Statistically significant differences were observed between Dr0 vs. Di0 and DrM vs. DiM (*p* = 0.000).

### 3.4. Plaque Index (PI)

**Figure 5:**
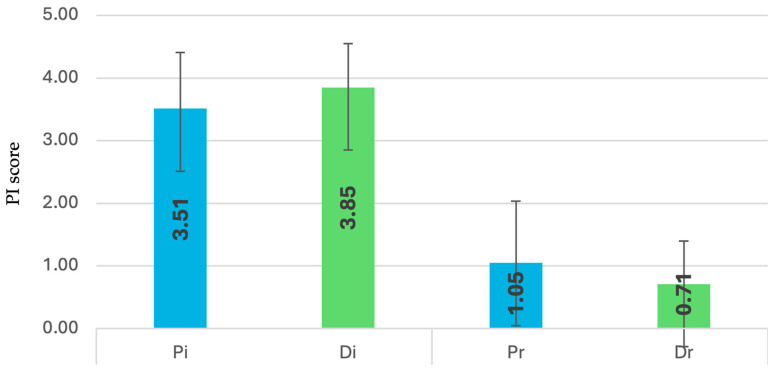
Changes in plaque index (PI) at baseline and 12 weeks after full-mouth disinfection in patients with and without depression.

Pi subgroup recorded the highest mean (Figure 5) at baseline (3.85 ± 0.70), whereas Pr had the highest at reevaluation (1.05 ± 0.99).

Tests indicated non-normal distribution and variance heterogeneity (*p* < 0.001), and Kruskal–Wallis analysis revealed significant differences between Pi vs. Pr, Pi vs. Di, and Dr vs. Di (*p* < 0.05).

Sex-based comparisons identified significant differences in Di (M) vs. Pi (M) (*p* = 0.004), along with multiple intergroup variations such as Pi (F) vs. Di (F) and Dr (F) vs. Pr (M) (*p* < 0.05).

In the depression group, both initial and reevaluation PI values were higher in SSRI-treated individuals (initial: 4.0 ± 0.6; reevaluation: 0.8 ± 0.7). A significant difference was observed between Dr0 vs. Di0 (*p* = 0.005)

## 4. Discussion

Our study demonstrated that the full-mouth disinfection (FMD) protocol effectively reduced plaque index (PI), bleeding on probing (BOP), probing depth (PD), and clinical attachment level (CAL) in patients with periodontal disease, including those also diagnosed with depressive disorder or stress. The research included 80 patients: 40 with isolated periodontal disease and 40 with coexisting depressive disorder. The mean age was 42.9 ± 10.2 years in the periodontal group and 41.8 ± 9.1 in the comorbid group, with minimal variation, hence age was not analyzed as a correlating factor—an approach consistent with previous findings showing PD and CAL are age-independent [20].

Recent studies confirm that debridement combined with oral disinfection enhances outcomes in advanced periodontitis (stages III–IV) by improving CAL and reducing PD and BOP [21]. However, in early stages (I–III), no significant differences were observed between SPR, FMS, and FMD protocols [22,23].

Gender analysis showed significant decreases in PD and PI after treatment across both sexes, regardless of depressive status, with no notable gender-based disparities, aligning with previous studies that found no link between periodontal indices and gender [16,24], despite contrasting reports suggesting men’s poorer oral hygiene habits [15,16].

Our results confirm that patients with depression presented more severe periodontal status at baseline and a reduced clinical attachment gain after therapy, supporting previous findings that psychological stress and depressive disorders negatively influence periodontal health and treatment outcomes [25,26,27]. Unlike many studies including smokers, our sample only involved self-declared nonsmokers. While CAL decreased significantly in non-depressed patients post-FMD, no improvement was seen in those with depression, similar to findings by Li et al. and Rosania et al. [28,29]. Though PD values improved in all patients post-FMD, initial PD scores were higher in depressed individuals, particularly when analyzed by gender.

Depressive disorders may impair periodontal health through neglected oral hygiene during depressive episodes [30]. However, systematic reviews have yielded mixed conclusions; one review of 15 studies found no link between depression and periodontitis progression [31,32]. Periodontitis is marked by attachment loss and immune dysregulation triggered by bacterial biofilm [3], and CAL variation is highly individual due to bone remodeling differences, including glucocorticoid-induced osteoporosis. Elevated cortisol, whether endogenous (stress-related) or exogenous, has been linked to bone loss and shifts in oral microbiota that promote periodontal inflammation [33,34].

Psychological status is thus a recognized risk factor for periodontitis. Meta-analyses show higher anxiety and depression scores in periodontal patients [35], and depression has been associated with reduced effectiveness of SRP treatment due to behavioral patterns like poor hygiene, smoking, alcohol use, and disrupted sleep or nutrition [8]. Our data showed significant reductions in PD, PI, and BOP across both patient groups post-FMD. However, CAL improved only in the non-depressed group.

Links between depression and periodontal disease are hypothesized to involve systemic inflammation, microbial translocation, or elevated lipopolysaccharides [36]. Cytokines from oral inflammation may cross the blood–brain barrier, contributing to neuroinflammation and immune suppression [37,38,39].

As it was specified before, the patients diagnosed with depression patients were divided based on SSRI use: 24 received pharmacologic treatment, 16 did not. SSRIs may alter periodontal disease progression and its response to therapy. Periodontal inflammation elevates cerebral cytokines, potentially reducing SSRI efficacy [40,41]. Conversely, inflammatory endotoxins may worsen depressive symptoms [36], and cortisol’s immunosuppressive effects may underlie this association [42].

Stress and depression modulate inflammatory mediators: glucocorticoids inhibit cytokines, while catecholamines enhance prostaglandin production, promoting tissue breakdown [32]. Elevated salivary cortisol and CAL have been linked to poor hygiene during depressive episodes [11], while PD has been associated with stress markers like α-amylase and β-endorphins [29]. Chronic stress perpetuates inflammation through macrophage and cytokine activation and T-helper cell imbalance, impairing healing [24].

SSRIs such as fluoxetine may reduce periodontal inflammation [27]. Depression alters the microbiome and disease severity, potentially interfering with antidepressant efficacy [31]. SSRIs have also been shown to lower oxidative stress and inflammatory markers, improving periodontal status [37]. In our study, SSRI treatment did not significantly alter periodontal indices, yet PD, PI, and BOP showed significant improvement post-FMD in both subgroups, consistent with previous research. Despite SSRIs’ anti-inflammatory potential, their impact on periodontal outcomes appears limited, and antidepressant use did not hinder FMD treatment efficacy.

This study has several limitations that should be considered when interpreting the results. First, depressive symptoms were not quantified using a standardized psychiatric scale, which may have affected the accuracy of group classification and the interpretation of clinical outcomes. Second, information on smoking status, medical history, and stress exposure was self-reported and may be subject to recall bias. Third, we did not assess biological markers such as salivary cortisol, pro-inflammatory cytokines, or microbiome profiles, which could have provided additional insights into the mechanisms linking depression and periodontitis. Finally, the relatively small sample size and short follow-up period (12 weeks) limit the generalizability of the findings.

Future research should include standardized psychiatric assessments, objective biomarkers, and larger, long-term multicenter studies to better understand the bidirectional relationship between depression, SSRIs, and periodontal healing.

## 5. Conclusions

– Full-mouth disinfection (FMD) proved effective in reducing probing depth (PD), bleeding on probing (BOP), and plaque index (PI) in the treatment of periodontal disease.
– Subjects with periodontal disease and comorbid depression or chronic stress exhibited significantly higher PD and PI levels compared to those without psychological comorbidities.
– The FMD protocol led to a consistent reduction in PD, BOP, and PI across all patients, with no differences observed between sexes.
– In patients with periodontal disease and depression or chronic stress undergoing selective serotonin reuptake inhibitor (SSRI) therapy, FMD treatment resulted in decreased BOP and PD values.
– Significant clinical attachment level (CAL) improvement occurred only in the non-depressed group, indicating depression may limit periodontal healing after non-surgical therapy.

## Figures and Tables

**Table 1:** Group and Subgroup Distribution.

Main Group	Subgroup—Initial	Subgroup—Reevaluation
Periodontitis (P)	Pi	Pr
Periodontitis + Depression (D)	Di	Dr

**Table 2:** Full-Mouth Disinfection Protocol.

Phase	Key Procedures
1. Assessment	PD, CAL, BOP, fluid sampling, plaque disclosure, motivation
2. FMD	Anesthesia, ultrasonic scaling, manual curettage, antiseptic rinses
3. Follow-up	Repeat clinical indices, oral hygiene reinforcement, re-instrumentation if needed

## Data Availability

All the data presented in this study are available within the article.

## Abbreviations

The following abbreviations are used in this manuscript:

PD	Probing Depth
CAL	Clinical Attachment Level
BOP	Bleeding on Probing
PI	Plaque Index
QH	Quigley Hein Plaque Index
FMD	Full-Mouth Disinfection
FMS	Full-Mouth Scaling
SRP	Scaling and Root Planing
Q-SRP	Quadrant-Based Scaling and Root Planing
FMDAP	Full-Mouth Disinfection with Air Polishing
SSRI	Selective Serotonin Reuptake Inhibitor
